# Neuralgia of the Fifth Nerve—Treatment

**Published:** 1895-10

**Authors:** B. Merrill Ricketts

**Affiliations:** Cincinnati


### SELECTIONS.

### Neuralgia of the Fifth Nerve—Treatment.

BY B. MERRILL RICKETTS, M.D., CINCINNATI.

The fifth nerve is more frequently the seat of pain than any other. The remoteness of its ganglion, together with the anatomical relations of it and its branches, renders it one of the most difficult to attack from a surgical point of view. The stretching, division and extirpation of one or more of its branches seem to have fallen short of the object sought for.

There is no pain which can equal in severity that associated with this nerve. Medication of all kinds seems to have fallen short of giving any very great relief, and, so far as I am able to determine, no case, until within a recent date, has ever been permanently relieved in this way. If in the removal of the ganglion of Gassa lies a remedy from a surgical standpoint, all will be glad.

There are but two operations which should receive any consideration: First in importance, the Hartley operation; second, the Langenbeck operation.

It is an utter impossibility to remove the Gasserian ganglion by the Rose operation upon the dead, much less upon the living body, where the field of operation is covered with blood, and the means insufficient to remove or even destroy that ganglion. It lies between the external and internal coats of the dura, which makes it all the more difficult to attack.

There are three principal objections to this operation: (1) Loss of the cornea, which occasionally ensues; (2) ankylosis of the lower jaw, which is always more or less present; (3) fistulæ. I would therefore exclude this operation from further consideration.

The Hartley operation is the most radical and scientific of all in this connection. It is by far one of the most difficult found in the domain of surgery. The hemorrhage which is sometimes severe, generally follows the detachment of the dura that it may be allowed to be pushed backwards; it may also result from the tearing away of the nerve from the dura, and it may, too, be the result of injury to the middle meningeal artery, which should always be ligated immediately upon its presentation to view, and it must necessarily be pushed back with the dura. After stripping the first and second branches to a point beyond the ganglion, they are divided, a section removed, and their ends tucked into the foramen ovale and rotundum respectively. In this way the ganglion is destroyed and all connection with the periphery obliterated.

The Langenbeck operation is next in importance to that of Hartley. I understand that the operation has been made about fifteen times by Langenbeck himself, and six times by Bernays, of St. Louis, one of which I saw the latter operator make. The technique is more difficult to understand than either of the other two, and it is this reason, perhaps, that has kept it from being popularized.

The operation is made by removing the first and second branches. The first, after having made a sub-periosteal resection of the lower jaw, which enables the foramen ovale to be reached and about two and a half inches of the nerve removed, the bone is reunited by a silver wire with the end protruding through the integument, so that it may afterward be removed. The second or infra-orbital branch is removed by dividing the nerve in the spleno-maxillary fissure, the knife having first been passed from before backward along the external wall of the orbit on a line with the meatus auditorius externus. The incision is first made at its exit, so that it may be extracted after this division is made. Quite a large hematoma within the orbit results, but at the end of a week is completely absorbed.

In all the cases thus far operated upon, so far as I am able to determine, a permanent cure has resulted.

I believe that the treatment of trifacial neuralgia is on the verge of a revolution, and, while I am sorry to see any of the glories of surgery lost, I hail with delight any remedy that will relieve these unfortunate people of their agony.—*Lancet Clinic.*

